# Rare complication of coupled VASER liposuction and Renuvion technologies: a case report

**DOI:** 10.1080/23320885.2023.2181175

**Published:** 2023-03-29

**Authors:** Moraya Alqahtani, Nehal Mahabbat, Khalid Fayi

**Affiliations:** a Consultant of Plastic & Reconstructive Surgery, Riyadh, SA; b Department of Plastic Surgery, King Faisal Specialist Hospital & Research Centre, Riyadh, SA

**Keywords:** Liposuction, J-Plasma, Renuvion

## Abstract

This study presents the case of a women, who underwent VASER-assisted liposuction of the abdomen coupled with Renuvion skin tightening by J-Plasma for skin retraction. She developed pain and moderate surgical emphysema. Radiological findings showed moderate subcutaneous emphysema. There were no signs of viscus perforation, or pneumothorax.

## Introduction

Since the second half of the twentieth century, there has been a focus on the human body, particularly on improving its appearance as a medium of self-identification and expression [1,2]. Liposuction has been regarded as a frontier in skin tightening when it comes to physique improvement [2]. The involvement of new technologies such as vibration amplification of sound energy at resonance (VASER), J-Plasma (helium plasma), and MicroAire have eased the surgical procedure with a reported lower rate of complications, and higher patient satisfaction [3,4]. Despite the many positive benefits of such technologies, new serious complications may still arise. There are no current reports on complications in the case of Renuvion, but data is still limited because this is a new technology [2,5–10]. This case report presents a rare complication associated with the utilization of combined VASER liposuction and Renuvion (J- Plasma) technologies.

## Presentation of case

**Figure 1.**
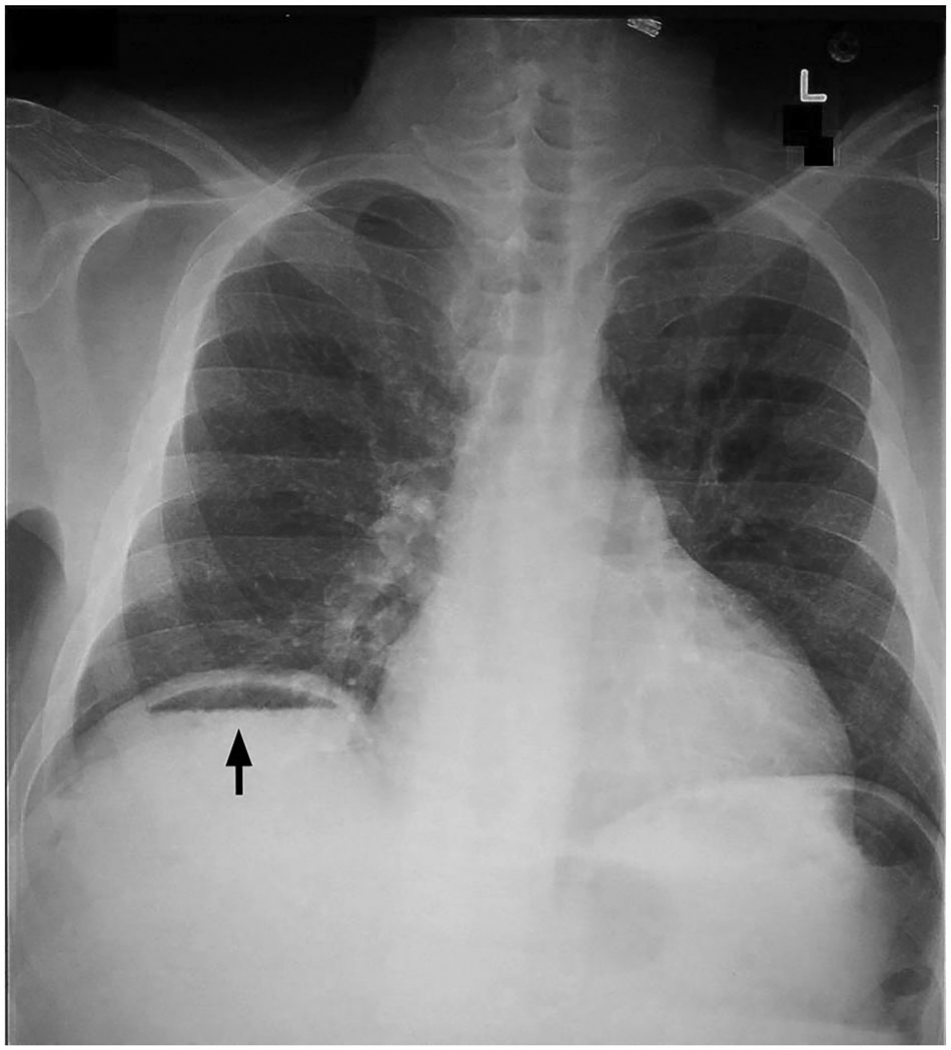
Chest x-ray of the patient shows the presence of air located under the diaphragm.

A single 34-year-old woman, not known to have any medical problems, BMI of this patient was 28 kg/m^2^, with medium abdominal wall thickness, she underwent VASER-assisted liposuction of the abdomen, flanks, and lateral thighs, coupled with Renuvion skin tightening by J-Plasma for skin retraction. On the 2nd postoperative day, the patient complained of pain in the right shoulder. The physical examination was unremarkable. The vitals and basic lab results were within the normal range. An X-ray of the abdomen showed pneumoperitoneum (Figure 1). A computerized tomography (CT) scan with contrast was also done. The radiological findings showed early postoperative changes related to anterior abdominal wall liposuctions with moderate subcutaneous emphysema. Mild pneumoperitoneum and pneumomediastinum were also observed. No signs of viscus perforation, extraluminal leakage of dense contrast material, or pneumothorax were noted. On the other hand, the visualized portion of the bowel was unremarkable. The patient was followed after 2 weeks, her symptoms started to improve in regards to pain, and recovered completely 1 month later. During the patient's recovery period pain killer were given, and instructions to come to ER with any worsening of her symptoms.

## Discussion

This case described a patient who developed moderate surgical emphysema at the abdominal wall postoperatively. The case of Lim and colleagues on a 48-year-old woman, who underwent similar VASER-assisted liposuction coupled with J-Plasma technology for the bilateral thighs at a cosmetic clinic, also reported pain after 10 h of the procedure. Consistent with the present case, a radiological examination of the 48-year-old woman revealed subcutaneous emphysema, observed on the neck, chest wall, abdominal walls, and thighs, as a rare complication of the mentioned combined technologies. Lim and colleagues described the use of VASER liposuction to impose complications such as subcutaneous emphysema [5–11]. According to the case report of Kim and co-workers, the mechanism of air entrapment was due to the negative pressure differential and gas accumulation around the fascial planes after air entry to the incised skin. This can result in gas accumulation, which can cause temporary and transient pain and crepitus. Complications related to J-Plasma are attributed to the volume of gas that was not ionized under the flap, which served as a coexisting air conditioner within its area of use [12,13]. Duncan (2019) mentioned that additional risks may also arise when using Renuvion’s J-Plasma technology in conjunction with other tools and if the patients had experienced previous surgical operations in the body part of interest before receiving the said technology [13,14]. Renuvion is still a new technology, so there is little reported evidence of its associated complications. Perhaps, the air seen in the diaphragm possibly resulting from J-Plasma utilization is another new and rare complication of Renuvion’s J-Plasma technology.

Previous studies revealed only a small thermal effect on tissue depth despite the application of multiple passes of the J-Plasma device [15]. Multiple passes are employed to maximize treating the tissue of interest [15]. Research on a live porcine model to determine the effect of Renuvion’s J-Plasma technology on soft tissue contraction also involved the utilization of multiple treatments passes [14]. At least one treatment pass (3 strokes per pass) of the Renuvion device was employed. Duncan (2019) mentioned that other studies require a series of three passes at multiple depths (numerous ray types of strokes) for an average body region of surgery [14].

Our proposed mechanism of this injury regarding subcutaneous emphysema is due to excessive residual helium gas with J- Plasma procedure, and in regards to pneumoperitoneum which is not necessarily associated with perforation of the intestine, this may accidentally happen during tumescent solution infiltration or liposuction with a small calibre canula, especially in females with thin muscle & posterior rectus sheath, however, CT abdomen didn’t show any bowel perforation.

We reported this case due to the limited reported cases of similar complications because it’s a new technology & particularly when used for skin tightening, it is considered a rare complication but with time & labeled use of this technology it might be more common and to keep surgeons aware of these possible complications.

We suggest being careful & gentle during infiltration of tumescent & liposuction especially with the female patient due to their thin abdominal wall to avoid similar complications.

Overall, this study highlights the importance of carefully monitoring the postoperative course and scheduling radiological examinations to avoid or mitigate serious consequences.

## Conclusion

Emphysema in the abdominal tissue and under the diaphragm are rare complications of J-Plasma technology along with liposuction, despite the overall good literature records on such technologies. Complications arising from its individual and combined use may eventually pose serious risks, implying the importance of using these techniques with greater caution. We suggest being careful & gentle during infiltration of tumescent & liposuction especially with the female patient due to their thin abdominal wall to avoid similar complications.

## Author note

The authors disclosed no finance-related engagement linked to this article.

## Ethical statement

Exempted from the IRB approval.

## Patient consent

Informed consent (written and verbal) was obtained from the studied patient.
